# Overexpression of the NEK8 kinase inhibits homologous recombination

**DOI:** 10.1016/j.dnarep.2025.103902

**Published:** 2025-10-08

**Authors:** Joshua L. Turner, Georgia Moore, Tyler J. McCraw, Jennifer M. Mason

**Affiliations:** Department of Genetics and Biochemistry, Clemson University, USA

**Keywords:** NEK8, homologous recombination, RAD51

## Abstract

Homologous recombination proteins maintain genome stability by repairing double strand breaks and protecting replication fork stability. Defects in homologous recombination results in cancer predisposition but can be exploited due to increased sensitivity to certain chemotherapeutics such as PARP inhibitors. The NEK8 kinase has roles in the replication response and homologous recombination. NEK8 is overexpressed in breast cancer, but the impact of NEK8 overexpression on homologous recombination has not been determined. Here, we demonstrate NEK8 overexpression inhibits RAD51 focus formation resulting in a defect in homologous recombination and degradation of stalled replication forks. Importantly, NEK8 overexpression sensitizes cells to the PARP inhibitor, Olaparib. Together, our results suggest NEK8 overexpressing tumors may be recombination-deficient and respond to chemotherapeutics that target defects in recombination such as Olaparib.

## 1. Introduction

A failure to repair double strand breaks (DSBs) can result in genome instability including genome rearrangements and cell death. Cells repair DSBs through two main pathways. Non-homologous end joining ligates the ends of the DSB back together. Homologous recombination (HR) uses a sister chromatid as a template resulting in repair of the DSB without the loss of genetic information. Inherited mutations in HR genes, including BRCA1 and BRCA2, are often linked to increased cancer risk [1,2]. On the other hand, HR-deficiency sensitizes cells to certain chemotherapeutic drugs including cisplatin and PARP inhibitors such as Olaparib [3,4]. Therefore, understanding how different alterations in cancer impact HR status has important implications for cancer therapy.

Proteins involved in HR have break-dependent and break-independent functions to maintain genome stability. At 5’ resected end of DSBs, RAD51 is loaded onto single-stranded DNA (ssDNA) by mediators including BRCA2 and RAD51 paralogs to form a nucleoprotein filament [5,6]. RAD51 filaments search for a homologous sequence to use as a template for repair. Once found, RAD51 catalyzes the strand exchange reaction by initiating pairing between the ssDNA and the complementary DNA sequence forming an intermediate known as a displacement loop, or D-loop [7,8]. RAD51 is removed from the D-loop structure exposing the 3’OH to allow for DNA synthesis across the region that contains the break. The D-loop is dissolved and DNA synthesis copies any missing information resulting in repair of the DSB without loss of information. At sites of stalled replication forks, HR proteins have several functions that do not require the presence of a DSB. RAD51 and RAD54 promote fork regression at stalled replication forks [9,10]. RAD51 binds to the regressed fork and is stabilized by several proteins including BRCA2, FANCD2, and FANCA to prevent nuclease-mediated degradation of nascent DNA [11]. Fork protection prevents nuclease degradation and cleavage by nucleases including MRE11, EXO1, DNA2, and MUS81 [12–15].

Homologous recombination is tightly controlled in cells to prevent unscheduled or inappropriate HR. Increased recombination is associated with increased chromosomal rearrangements and has been linked to chemotherapeutic resistance [16]. RAD51 is tightly controlled by proteins that promote nucleoprotein filament assembly and stability including BRCA2 and RAD51 paralogs or disrupt RAD51-DNA interactions including RAD54 and FBH1 [17–20]. RAD51 is also controlled by post-translational modifications that alter RAD51-DNA binding and subcellular localization. RAD51 phosphorylation on residues Ser14 and Tyr13 by PLK1, CK2, and CHK1 promote homologous recombination [21,22]. In contrast, the kinase c-Abl phosphorylates RAD51 on Tyr315 and Tyr54 inhibiting RAD51 nucleofilament formation and nuclear localization [23,24]. Outside of phosphorylation, ubiquitination of RAD51 by FBH1 negatively regulates HR by inhibiting rebinding of RAD51 to DNA [17]. Other HR proteins are also regulated by post-translational modifications including RAD54 and BRCA2 [25–27].

Never in mitosis gene A (NIMA) related kinase 8 (NEK8) is a member of a serine/threonine kinase family involved in G2/M progression, DNA repair, centromere localization, and cilia development [28–30]. In primary cilia development, NEK8 regulates cilia length as well as centrosome recruitment [30]. Dominant and recessive mutations in NEK8 leads to juvenile cystic kidney disease (JCK) in mice and nephronophthisis in humans [31,32]. NEK8 plays a role in the G2/M checkpoint by regulating the expression of enzymes such as CDK1 and Cyclin B1 [33]. More recent studies have shown that NEK8 has several roles in S-phase, specifically in DNA repair. NEK8 has been shown to directly interact with ATR in the ATR-CHK1 signaling pathway, regulating the double strand break response and new origin firing [33]. NEK8 promotes RAD51 focus formation at sites of DSBs, HR, and replication fork protection [34].

NEK8 overexpression has been found in a high percentage of non-specific invasive breast cancer [35]. Overexpression of NEK8 in breast cancer carcinomas was found to be involved in increased growth rate, colony formation, and proliferation [36]. Overexpression of JCK mutated NEK8 reduced actin expression as well as significantly increasing G2/M related protein expression [35]. The role of NEK8 overexpression on tumor progression was associated with its interactions with β-catenin in the Wnt/β-catenin pathway leading to increased cancer cell motility [36]. However, the effects of increased NEK8 expression on homologous recombination have not been explored.

Here, we show that NEK8 overexpression inhibits RAD51 foci formation resulting in a defect in homologous recombination and increased nascent strand degradation of stalled replication forks. Finally, NEK8 overexpression sensitizes cells to the PARP inhibitor, Olaparib. Together, our results indicate NEK8 overexpression may result in HR-defective tumors that will respond to treatment with PARP inhibitors.

## 2. Methods

### 2.1. Cell culture and drug treatment

hTERT immortalized RPE-1 cells were grown in DMEM/F12 50:50 (Corning, 10–092-CVR) with 10 % FBS (Atlas Biologicals, S10350H). U2OS cells were grown in DMEM (Gibco, 11965–092) with 10 % FBS. Cells were incubated at 37 ^◦^C in 5 % CO_2_. Hydroxyurea (Fisher Scientific, AAA1083106) was suspended in water. Olaparib (Thermo Scientific, 466292500) was suspended in DMSO. Cells were treated with the indicated concentrations.

### 2.2. Plasmid transfection

Cells were transfected with MYC-tagged NEK8 [32]. Cells were seeded at 200,000 cells in 6-well plates and transfected with Lipofectamine 3000 (Fisher Scientific, L3000001) using manufacturer’s instructions. Transfection efficiency was determined by transfecting cells with an mCherry containing plasmid. 48 h post transfection cells were analyzed by flow cytometry using a Beckman Coulter CytoFLEX S and analyzed using FCS Express 7 version 7.14.0020. Overexpression of NEK8 was verified by western blot.

### 2.3. siRNA transfections

RPE1 cells were seeded at 100,000 cells in 6-well plates and allowed to adhere for 24 h. Cells were transfected with Lipofectamine RNAiMAX (Fisher Scientific, 13778150) using manufacturers’ instructions. siRNAs against FANCA (Dharmacon, J-019283–07–0020, 75 nM) and four NEK8 siRNAs (Dharmacon, J-004866–05, J-004866–06, J-004866–07, J-004866–08, 75 nM) were pooled together. The All-stars negative control (Qiagen) was used as a negative control siRNA. The siRNA sequences are as follows:

siFANCA 5’ GGGCCAUGCUUUCUGAUUU

siNEK8 5’ AGACAAAGCCCUUAUGAUC

GUAAUUCCCUGCUGGAGGA

GCGAAAGGCUGACCAGAAG

GGGCAGAGAGCGAAGUGUA

### 2.4. Immunofluorescence

Cells were seeded at 40,000 cells in a 12-well plate containing 22 mm circular cover slips. Cells were treated with 2 mM HU or 10 μM Olaparib for 8 h. Cells were permeabilized (20 mM HEPES, ph7.4, 3 mM MgCl_2_, 50 mM NaCl, 0.5 % Triton X-100) for 10 min prior to fixation with 3 % PFA, 3.4 % sucrose. For PCNA staining, cells were fixed with ice-cold methanol. Slides were stained with primary antibodies overnight at 4 ^◦^C. Primary antibodies used in this study were PCNA (ABCAM, ab18197, 1:1000) and rabbit RAD51 (Pacific Immunology; 1:1000) [37]. Cells were washed 3 times with 1X PBS followed by incubation with Alexa Fluor-conjugated secondary antibodies (Invitrogen, 1:1000) for 1 h at room temperature. Slides were washed 3 times for 5 min in 1X PBS followed by 2 min each in 70 %, 95 % and 100 % ethanol. Coverslips were allowed to air dry and mounted with vectashield containing DAPI. Images of 100 PCNA positive nuclei were acquired at 60x magnification on a Zeiss Imager M2 epifluorescence microscope with an Axiocam 503 mono camera. The number of RAD51 foci in PCNA-positive cells was quantified using Image J software.

### 2.5. QIBC S-phase analysis

**Fig. 1.**
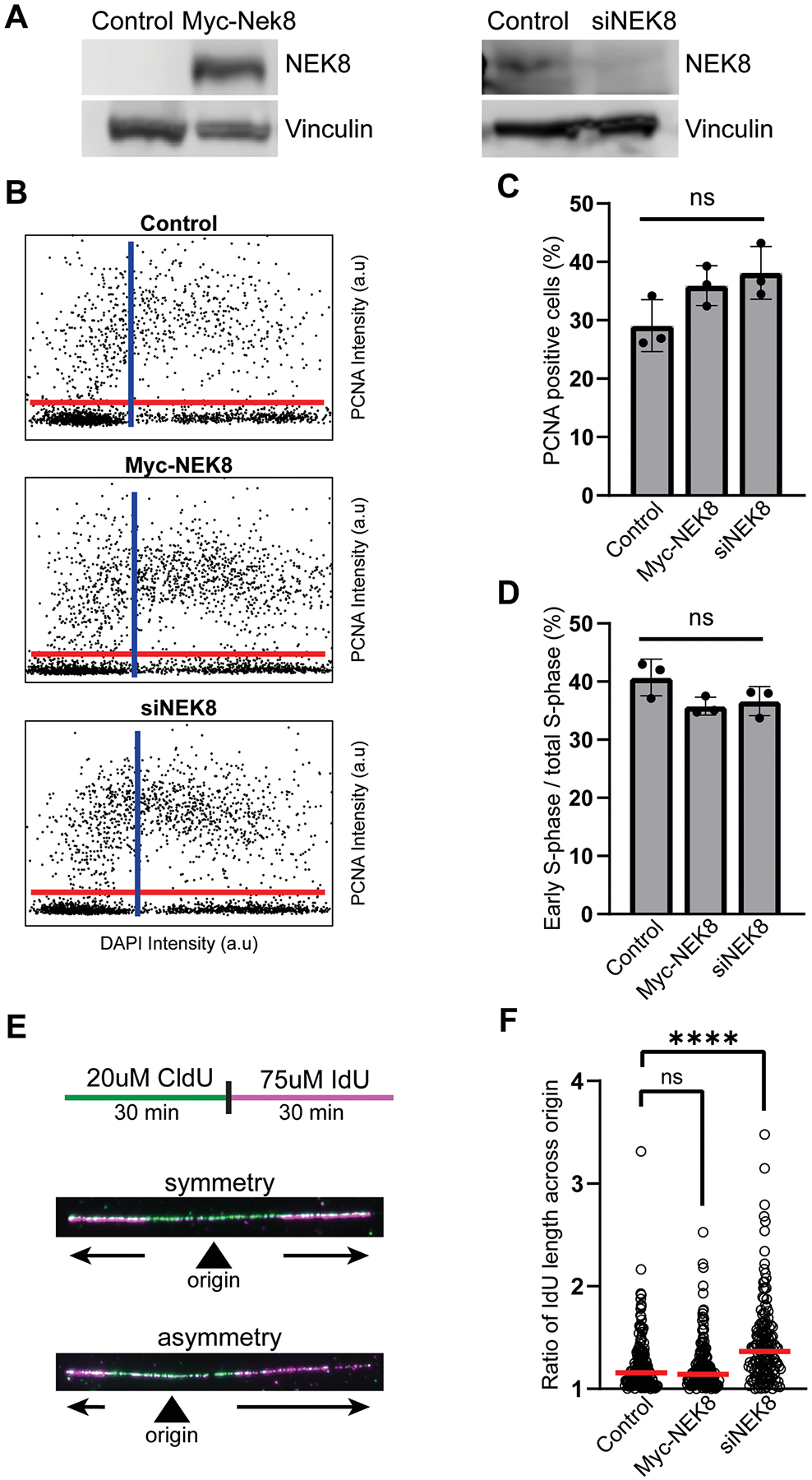
NEK8 overexpression does not affect replication dynamics (A) Western blot showing NEK8 overexpression with Vinculin loading control and NEK8 knockdown with Tubulin loading control (B) QIBC based images of Total DAPI intensity (X axis) with average PCNA (Y axis). Above the red line are cells considered to be in S-phase and cells to the left of the blue represent pre-replication or early S-phase. (C) Bar graph showing percentage of cells in S-phase (PCNA positive), black dots indicate individual replicates with standard error bars. (D) Bar graph of percent early S-phase to total S-phase, black dots indicate individual replicates with standard error bars. (E) Schematic of replication fiber assay pulsing conditions with representative images depicting fork symmetry and fork asymmetry. **(F)** Dot plot showing ratio of IdU lengths per origin with red line representing the mean. ~150 origins per condition, N = 3, ****p< 0.0001 ANOVA; Tukey HSD.

Slides were prepared as described above. 20x automated images were taken using Molecular Devices ImageExpress Micro 4 and analyzed for PCNA and DAPI signal intensity using the ImageExpress proprietary MetaXpress software (version 6.7.2.290). Total DAPI intensity was graphed against average PCNA intensity using Graphpad Prism (version 10.2.3). Early S-phase was determined by the percentage of PCNA positive cells that contained N2 equivalent DAPI intensity values as shown in Fig. 1B.

### 2.6. Replication fiber analysis

Cells were seeded at 40,000 cells in 12-well plates and allowed to adhere for 24 h. Cells were sequentially pulsed for 20 min with 20 μM CldU and 75 μM IdU. Cells were washed 2X with 1X PBS and treated with 2 mM hydroxyurea for 5 h before collecting by trypsinization. Untreated controls were collected by trypsinization immediately following the IdU pulse. Cells were resuspended in ice cold 1X PBS at a concentration of 40,000 cells/ml. Replication fibers were prepared as previously described [11]. Slides were stained with mouse BrdU (BD, 347580, 1:50) to label IdU and rat BrdU (Abcam, ab6326, 1:200) to label CldU. Slides were washed with 1X PBS followed by incubation with Alexa Fluor-conjugated antibodies (Invitrogen, 1:1000) Images were acquired at 60x magnification using the Zeiss epifluorescence microscope. The length of at least 150 replication tracts was measured using ImageJ.

### 2.7. Survival assay

Cells were plated in triplicate at 2000 cells per well in black walled 96-well plates (Corning, 3606). Cells were mock treated with DMSO or Olaparib for 4 days before staining with propidium iodide and Hoechst 33342. Live cells were counted and analyzed using Molecular Devices ImageExpress Micro 4 and proprietary MetaXpress software.

### 2.8. DR-GFP assay

U2OS cells were transfected with a pDRGFP plasmid, mCherry pCAGGs, and either empty vector (pCAGG, negative control) or pCBA SceI plasmids [38]. pDRGFP (Addgene #26475) and pCBASceI (Addgene #26477) were gifts from Maria Jasin [4,38]. mCherry pCAGGs (Addgene # 41583) was a gift from Phil Sharp [39]. 24 h post transfection fresh media was added to each well. 48 h post transfection cells were collected using trypsinization. mCherry positive cells were gated and percentage of cells positive for GFP was determined (i.e., HR positive). Flow cytometry was conducted as described above.

### 2.9. Nuclear fractionation

Cells were seeded at 600,000 cells in a 10 cm dish and allowed to adhere for 24 h. Cells were then treated with 2 mM HU or 10 μM Olaparib for 24 h. Cells were collected by trypsinization. Nuclear and cytoplasmic fractions were isolated using the nuclear and cytoplasmic extraction kit (Thermo Scientific, 78833) following manufacturer conditions. Protein levels were examined by western blotting.

### 2.10. Western blotting

Whole cells extracts were prepared by lysing cells (10^6^/100 ml) in Laemmli buffer (62.5 mM Tris-HCl, ph6.8, 2 % SDS, 10 % glycerol, 5 % 2-mercaptoethanol, and 0.002 % bromophenol blue) and boiled for 10 min. Extracts were separated on an SDS-Page gel and transferred to PVDF membrane. Membranes were stained with RAD51 (Novus Biologicals, NBP2–75640, 1:1000), Vinculin (Cell signaling, 13901S, 1:1000) and NEK8 (kind gift from David Bier, 1:1000) [40]. For the cell fractionation western, we stained for Histone H3 (Novus Biologicals, NB500–171, 1:1000) and α-Tubulin (Novus Biologicals, NB100–690, 1:1000). Membranes were washed with 1X TBS + 0.1 % Tween-20 twice for 10 min. Membranes were incubated with HRP secondary antibodies (LI-COR, 926–80011/10, 1:2000). Membranes were washed with 1X TBS + 0.1 % Tween-20 twice for 10 min. Membranes were incubated with WesternSure Premium chemiluminescent substrate (LI-COR, 926–95000). Blots were imaged and analyzed using the LI-COR C-DiGit imager and proprietary Image Studio software version 5.2.5.

### 2.11. Statistical analysis

All experiments were performed at least three independent times. Statistical analysis was performed using Graphpad Prism. Significance was calculated using an ANOVA followed by Tukey HSD for RAD51 foci, fork deprotection, and DR-GFP assays. To calculate the significance in the survival assay we conducted an ANOVA followed by the Holm-Ŝídáks multiple comparisons test. Statistical significance was determined by a *p*-value below 0.05.

## 3. Results

### 3.1. NEK8 overexpression does not alter replication fork progression

NEK8 regulates the cell cycle by promoting activation of checkpoint enzymes such as CDK, Cyclin A, and CHK1 [33,41,42]. Direct interactions with ATR also implicated NEK8 in regulating replication dynamics and S-phase progression [33]. We wanted to determine how S-phase populations in NEK8 overexpressing cells compared to cells depleted of NEK8. To test this, we transfected RPE1 cells with Myc-tagged NEK8 (Myc-NEK8) or siRNAs targeting NEK8 (siNEK8) (Fig. 1A). A transfection efficiency of 80 % was verified by flow cytometry using mCherry expression (Figure S1). First, we examined if NEK8 overexpression or depletion impacted cells in S phase by measuring PCNA intensity in cells based on total DNA content using QIBC [43]. We observed a small but insignificant increase in the total S phase population (Figs. 1B, 1C). Furthermore, we examined early S phase cells and found no significant difference in the percentage of cells in early S phase between control, Myc-NEK8 and siNEK8 cells (Fig. 1D). Thus, NEK8 overexpression or depletion does not alter S phase progression in unperturbed cells.

NEK8 is critical for replication fork progression and loss of NEK8 results in a significant increase in fork stalling and/or collapse leading to fork asymmetry [33]. We wanted to determine if NEK8 overexpression resulted in defects in replication fork progression in unperturbed cells. To test this, we measured the length of sister replication forks (i.e., bi-directional forks that are derived from a single origin) [44]. We sequentially pulsed cells with thymidine analogs CldU and IdU for equal lengths of time and measured IdU lengths of the left and right IdU tract (Fig. 1E). Under unperturbed conditions, replication forks initiating from a single origin will move at approximately the same speed resulting in symmetrical right and left forks. However, if one of the replication forks stalls or collapses this will result in a shorter IdU tract on one of the sister forks resulting in fork asymmetry (i.e., higher ratio of long:short tract). As published previously, sister replication forks in siNEK8 cells exhibited a significant increase in asymmetry as indicated by a higher longer to shorter IdU ratio compared to control cells (Fig. 1F) [33]. In contrast, replication forks in NEK8 overexpressing cells did not exhibit fork asymmetry and the IdU ratio did not significantly differ from controls. Thus, NEK8 overexpression differs from NEK8 depletion and does not result in fork asymmetry due to increases in replication fork stalling and/or collapse. Together, these results suggest NEK8 overexpression does not impact cell cycle progression or replication fork progression in unperturbed cells.

### 3.2. NEK8 overexpression inhibits RAD51 focus formation

**Fig. 2.**
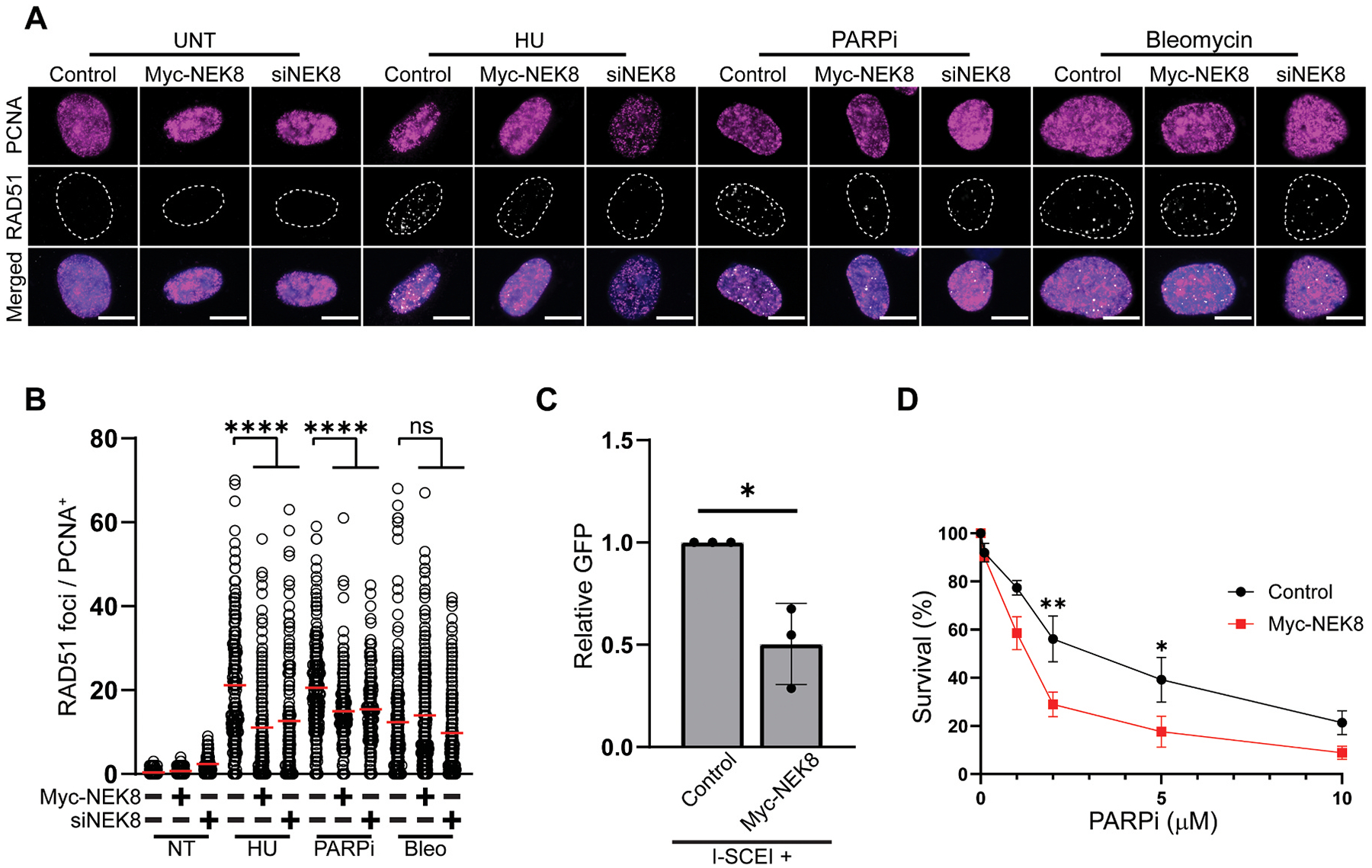
NEK8 overexpression inhibits homologous recombination (A) Representative images of PCNA (magenta), RAD51 foci (white), and channels merged with DAPI. Scale bar = 10 μm. (B) Dot plot showing RAD51 focus counts for > 150 cells per condition, red bar indicates sample mean. n = 3, **** *p*< 0.0001 ANOVA; Tukey HSD. (C) Bar graph of percent GFP positive cells relative to the I-SCEI positive control per experiment. N = 3, error bars = SD, ***p< 0.001 ANOVA; Tukey HSD. (D) Survival curve of control and Myc-NEK8 cells after treatment with PARPi for 4-days, N = 3, error bars = SEM.

Previous studies have indicated that NEK8 is required for proper RAD51 localization to the sites of DSBs [34]. At resected DNA ends, RAD51 forms nucleoprotein filaments that can be visualized by immunofluorescence microscopy. We determined if NEK8 overexpression impacted RAD51 localization by measuring RAD51 focus formation after treatment with hydroxyurea (HU), bleomycin, or the PARP inhibitor, Olaparib (hereafter PARPi). We depleted NEK8 (siNEK8) to determine how NEK8 overexpression compared to NEK8 knockdown. In control cells, treatment with HU, PARPi, and bleomycin significantly increased RAD51 focus formation (Fig. 2A). Compared to the empty vector control, Myc-NEK8 overexpression resulted in a 1.88-fold decrease in RAD51 focus formation after HU and a 1.38-fold decrease in RAD51 focus formation after treatment with PARPi (Figs. 2B, S2A). Compared to the control, siNEK8 resulted in a 1.67-fold decrease after HU and a 1.34-fold decrease after treatment with PARPi. There was no significant difference in the average number of RAD51 foci between overexpression or knockdown of NEK8. Interestingly, neither Myc-NEK8 nor siNEK8 had a significant difference in RAD51 foci compared to control cells following treatment with bleomycin. Together these results indicate that NEK8 overexpression and NEK8 knockdown inhibit RAD51 localization to double strand breaks induced by PARPi and HU, but not bleomycin. These results suggest that NEK8 plays a role in promoting RAD51 to a subset of double strand breaks.

### 3.3. NEK8 overexpression inhibits HR

The reduction of RAD51 focus formation in response to HU and PARPi treatment suggests that NEK8 overexpression may inhibit HR. To test this, we measured HR efficiency using the DR-GFP assay in U2OS cells [38]. Compared to the empty vector control, Myc-NEK8 overexpression resulted in a 2-fold decrease in GFP positive cells (Fig. 2C). These results suggest that the defect in RAD51 localization in NEK8 overexpressing cells is sufficient to inhibit HR.

A defect in HR, such as BRCA2-deficiency, results in increased sensitivity to PARP inhibitors [2]. We tested if Myc-NEK8 overexpression increases sensitivity to PARP inhibitors by treating cells with PARPi. We found that Myc-NEK8 overexpressing cells had decreased survival when treated with PARPi compared to control cells (Fig. 2D). Together, these results indicate NEK8 overexpression increases sensitivity to PARP inhibition consistent with a defect in HR.

### 3.4. NEK8 overexpression promotes replication fork degradation

**Fig. 3.**
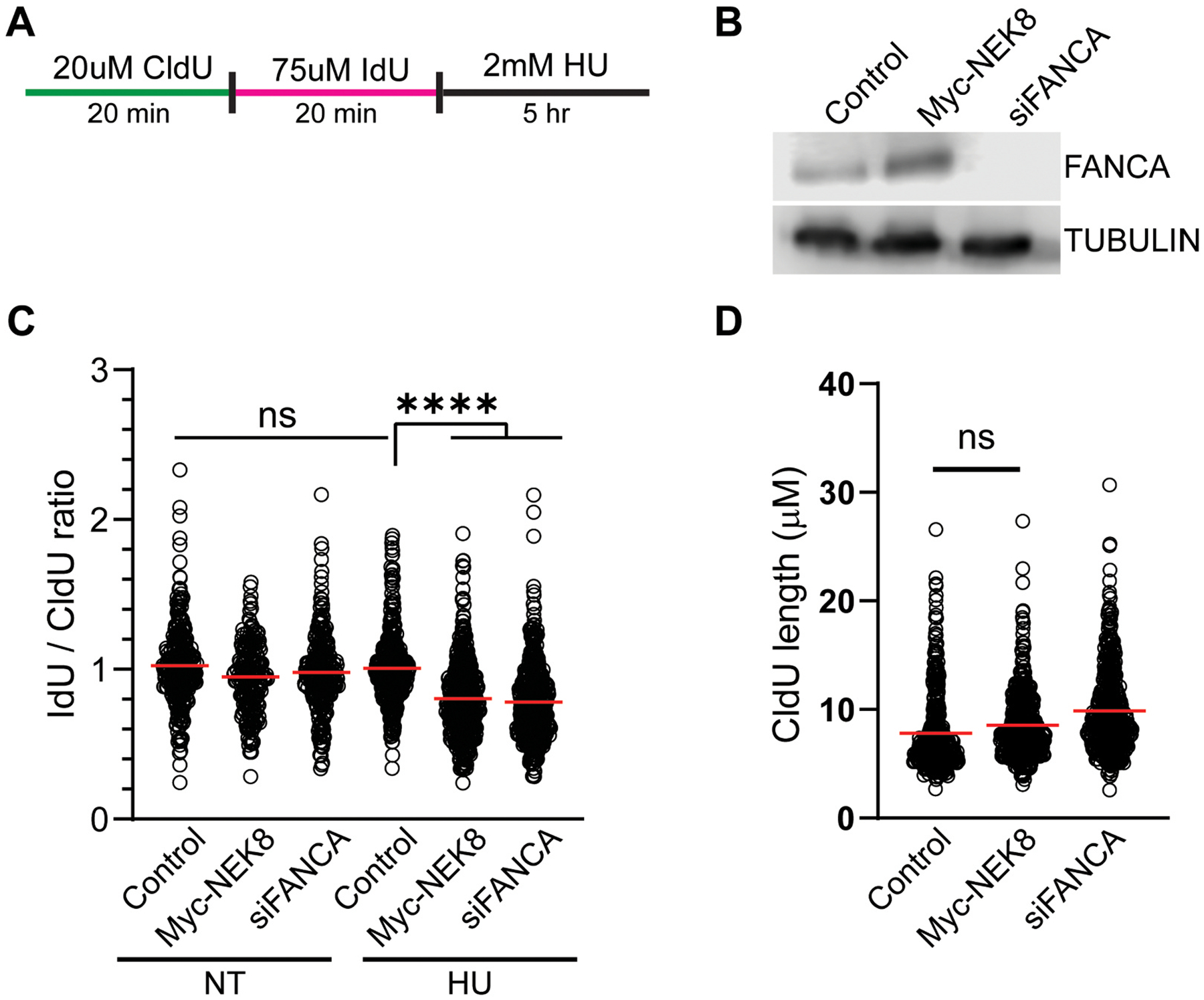
NEK8 overexpression promotes fork deprotection. (A) Schematic to measure fork deprotection using the replication fiber assay. (B) Western blot depicting FANCA protein levels under indicated treatment, Tubulin used for loading control. (C) Dot plot depicting ratio of IdU / CldU tract lengths, red line represents the mean. (D) Dot plot depicting CldU lengths of each sample, red line represents the mean. > 200 fibers per condition, N = 3, ****p< 0.0001 ANOVA; Tukey HSD.

At stalled replication forks, RAD51 protects nascent DNA from degradation by nucleases such as MRE11 [45,46]. Therefore, we determined if the decrease in RAD51 localization in NEK8-overexpressing cells results in nascent strand degradation using the replication fiber assay [47]. After sequentially pulsing control and Myc-NEK8 cells with thymidine analogs chlorodeoxyuridine (CldU) and iododeoxyuridine (IdU), cells were treated with hydroxyurea (Fig. 3A). If replication forks undergo nucleolytic degradation, IdU tracts will be degraded leading to a reduction of the IdU/CldU ratio (Figs. 3B, 3 C, S2B). As a control, we measured replication tract lengths in cells depleted of the fork protection protein, FANCA, and observed a significant decrease in the IdU/CldU ratio (0.995 in controls vs. 0.777 in FANCA-depleted cells) [11]. Myc-NEK8 expressing cells resulted in a significant decrease in IdU/CldU ratio from 0.995 in control cells to 0.801 (Fig. 3C). Additionally, we did not observe a significant reduction in CldU lengths indicating the reduction in the IdU/CldU ratio observed in NEK8 overexpressing cells is not due to a reduction in replication fork speed (Fig. 3D). Together, these results indicate that overexpressing NEK8 leads to increased nascent strand degradation at stalled replication forks.

### 3.5. Changes in RAD51 focus formation are not due to a reduction in nuclear RAD51

**Fig. 4.**
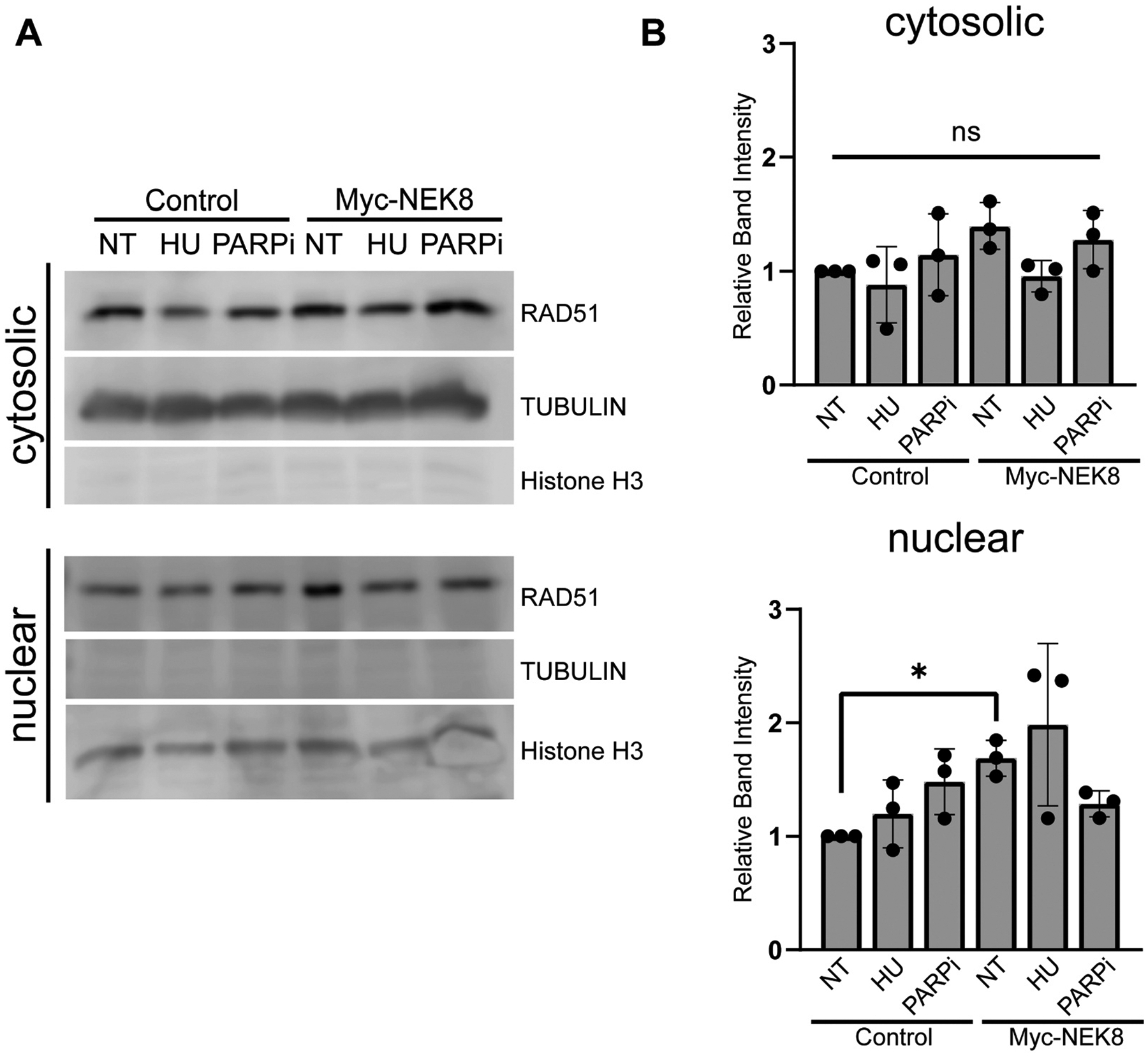
NEK8 OE promotes marginal increase in nuclear RAD51 localization. (A) Western blot depicting RAD51 protein levels in cytosolic (Top) and nuclear fractions (Bottom). Tubulin (cytosolic) + Histone H3 (nuclear) were used as loading controls. (B) Bar graphs depicting relative band intensity of RAD51 in cytosolic (Top) western blots and nuclear fractions (Bottom) western blots. N = 3, error bars = SD, *<0.05 ANOVA; Tukey HSD.

It is unclear how NEK8 regulates RAD51 focus formation. It has been shown that post-translational modification of RAD51 can influence its ability to traverse into the nucleus [23]. We determined if loss of RAD51 focus formation is due to a reduction in RAD51 protein levels or decreased RAD51 nuclear localization. To test this, we isolated cytosolic and nuclear protein extracts and analyzed RAD51 expression by western blot (Fig. 4 A). Myc-NEK8 expressing cells showed no significant difference in cytosolic localization of RAD51 compared to control cells regardless of treatment (Fig. 4B). In untreated cells, MYC-NEK8 overexpression resulted in a 1.68-fold increase in nuclear RAD51 localization. We also observed a 1.785-fold increase in nuclear RAD51 localization in Myc-NEK8 cells compared to controls after HU treatment indicating NEK8 overexpression leads to a slight, but significant increase in nuclear RAD51 localization. This result indicates the reduction of RAD51 focus formation in Myc-NEK8 overexpressing cells is not due to a reduction in RAD51 nuclear localization.

## 4. Discussion

NEK8 promotes RAD51 focus formation and activation of the ATR mediated stress response [34]. However, studying overexpression of NEK8 may provide important molecular insight into how up-regulation of NEK8 contributes to invasive breast cancer [36]. We demonstrated NEK8 overexpression inhibits HR, and the inhibition is likely due to a reduction in RAD51 localization to the sites of breaks. In this study, we demonstrated that overexpression of NEK8 caused a significant reduction in RAD51 focus formation after treatment with HU or PARPi similar to knockdown of NEK8. We also found that NEK8 overexpression or depletion does not inhibit RAD51 focus formation after bleomycin treatment, indicating that NEK8 may function to regulate RAD51 localization to a subset of breaks. Consistent with this, we observed a 2-fold decrease in HR efficiency in NEK8 overexpressing cells. Given PARPi and HU both cause replication-associated damage, we propose NEK8 regulation primarily functions at damage during replication. Consistent with this, we also found NEK8 overexpression resulted in nascent strand degradation of stalled replication forks.

In NEK8 overexpressing cells, we observed HR defects and replication fork degradation similar to phenotypes observed in NEK8-deficient cells [33,34]. However, we observed fork asymmetry in NEK8-depleted cells, but not upon NEK8 overexpression, indicating NEK8 overexpression is not phenocopying NEK8-deficiency in cells [33]. NEK8 is not the only kinase that exhibits similar phenotypes upon depletion and overexpression. The Polo-like Kinase 1 (PLK1) is required for efficient DNA end resection to promote homologous recombination [48,49]. Overexpression of PLK1 inhibits homologous recombination by an unknown mechanism [50].

The mechanism by which NEK8 overexpression inhibits HR is not clear. Given NEK8 overexpression leads to a defect in replication fork stability and a defect in HR, we speculate NEK8 is likely playing a role in regulating RAD51 loading at sites of damage. One possibility is NEK8-mediated phosphorylation of RAD51, or other HR proteins is resulting in reduced RAD51 nucleoprotein filament formation. Phosphorylation of RAD51 at Tyr54 and Tyr315 negatively regulate RAD51 activity [23]. It is unlikely an increase in RAD51 phosphorylation at Tyr54 results in a defect in RAD51 focus formation in NEK8 overexpressing cells. Phosphorylation of RAD51 at Try54 inhibits the import of RAD51 to the nucleus and we did not observe a decrease in RAD51 protein levels in the nuclear fraction in NEK8 overexpressing cells [23]. In contrast, we observed an increase in nuclear localization of RAD51 while maintaining a reduction in filament formation. Another possibility is NEK8 is leading to increased phosphorylation of other HR proteins such as BRCA2. For instance, CDK phosphorylates BRCA2 at Ser3291 to disrupt the interaction between the C-terminal region of BRCA2 and RAD51 [25]. NEK8 regulates Cyclin A-associated CDK activity, but it is not clear if NEK8 has roles in other CDK-dependent functions [33]. NEK8 overexpression may be leading to mis-regulation of the step of HR regulated by NEK8. Another possibility is NEK8 has a role in both positive and negative regulation of HR. A future area of research will determine how NEK8 regulates HR and if the impact on HR in NEK8-deficient cells and NEK8-overexpression is due to distinct mechanisms.

In human cells, 11 NIMA-related kinases have been identified with functions in mitosis, primary cilia, and DNA damage response and repair. NEK kinases mediate mitotic progression (NEK2, NEK6, NEK7, and NEK9), regulate replication and proliferation (NEK3, NEK8) and function in the G2/M checkpoint (NEK1, NEK10, NEK11) in both unperturbed and in response to genotoxic stresses [51–57]. Several NEK kinases besides NEK8 have been shown to be involved in the DNA damage response and in promoting DNA repair [58]. NEK1 phosphorylates RAD54 to restrict HR activity to the G2 phase of the cell cycle [27]. NEK2 is required for RAD51 focus formation in response to irradiation [59]. Thus, like NEK8, the other NEK kinases play critical roles in modulating cell cycle and DNA damage responses.

Mis-regulation of NEK kinases is observed in a wide variety of cancers including breast cancer, gastric cancer, pancreatic cancer, and colon cancer [35,60–62]. NEK8 overexpression plays a direct role in the proliferation and invasiveness of many breast cancers [36]. Notably, up-regulation of NEK kinases including NEK2, NEK5, NEK6, and NEK7 also play a role in the progression of breast cancer [63–67]. Up-regulation of the kinase TLK1 in prostate cancer resulted in increased phosphorylation and activation of NEK1 and preventing NEK1 activation delayed cancer progression [68]. Thus, NEK kinases including NEK8 play critical roles in maintaining genome stability suggesting mis-regulation of NEK kinases promotes tumorigenesis.

Our results here have implications for the impact of NEK8 overexpression in cancer. Knocking down NEK8 was effective in reducing tumor proliferation indicating NEK8 is promoting proliferation of breast cancer cells [35]. Here, we demonstrate that NEK8 overexpression results in a decrease in RAD51 focus formation and HR deficiency. Cancer cells defective in HR (e.g. BRCA2 deficient breast cancer) are hyper-sensitive to PARP inhibitors such as Olaparib [69]. Consistent with a defect in HR, we found NEK8 overexpression increased sensitivity to Olaparib. Thus, non-specific breast cancers containing increased NEK8 expression may be sensitive to PARP inhibitors. Future work will need to be conducted to determine how breast cancer cells with elevated NEK8 respond to PARP inhibition.

## Supplementary Material

1

## Data Availability

No data was used for the research described in the article.

## Appendix A. Supporting information

Supplementary data associated with this article can be found in the online version at doi:10.1016/j.dnarep.2025.103902.

## Abbreviations:

DSBs: double strand break
HR: Homologous Recombination
ssDNA: single-stranded DNA
HU: Hydroxyurea
JCK: juvenile cystic kidney disease
NIMA: Never in Mitosis gene A
NEK8: NIMA related kinase 8
CldU: chlorodeoxyuridine
IdU: iododeoxyuridine
