# Supplementary material for: Overexpression of the NEK8 kinase inhibits homologous recombination
Source: DNA Repair (Amst). Author manuscript; Available in PMC 2026 Jul 2. (PMC13325567; doi:10.1016/j.dnarep.2025.103902)
Supplement: 1 [file NIHMS2184621-supplement-1.docx]

**Supplemental Information**


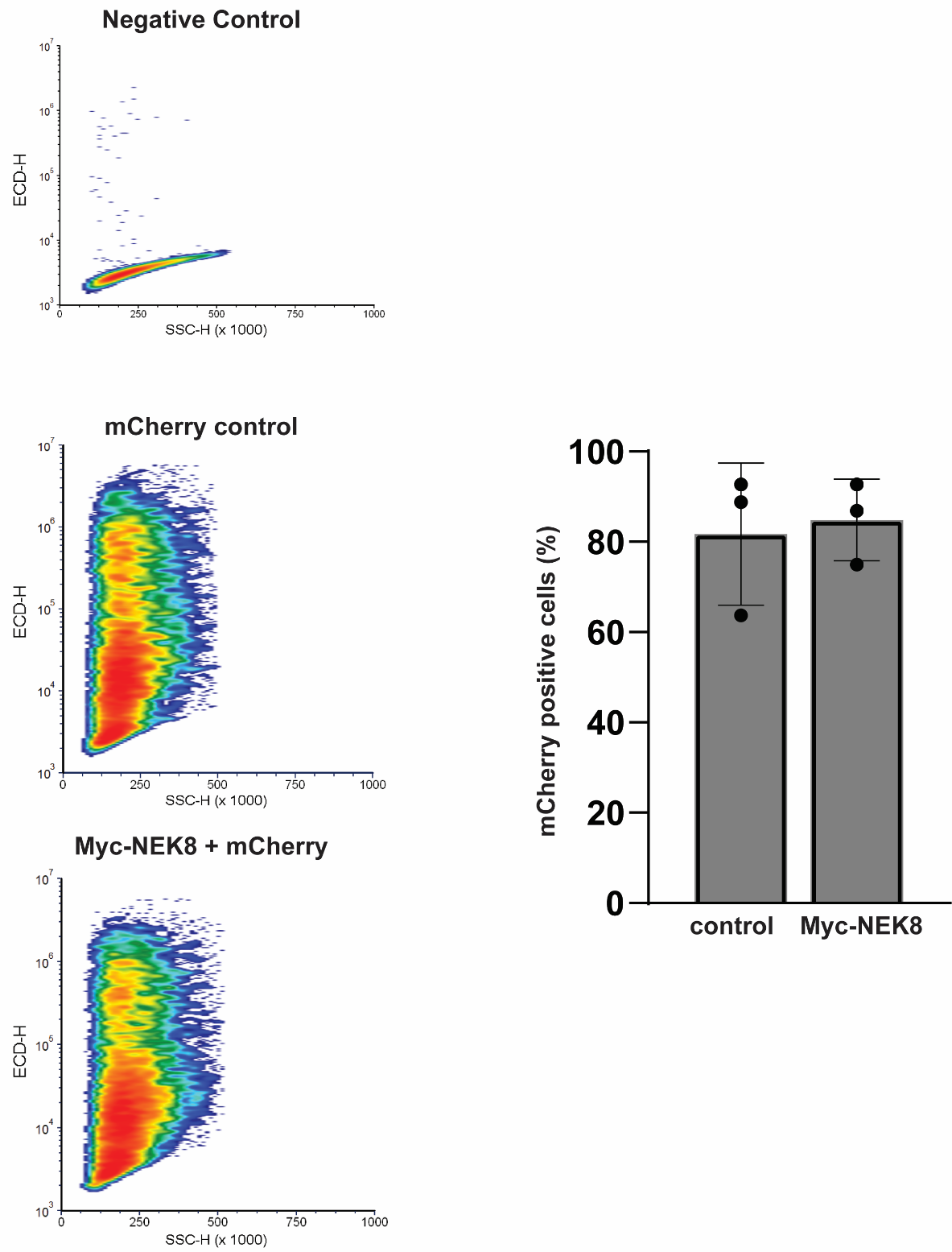


**Figure S1. Plasmid transfection efficiency in RPE1 cells.** Representative density plots for mCherry expression in RPE1 cells and the corresponding bar graph where black dots indicate individual replicate means with standard error lines.


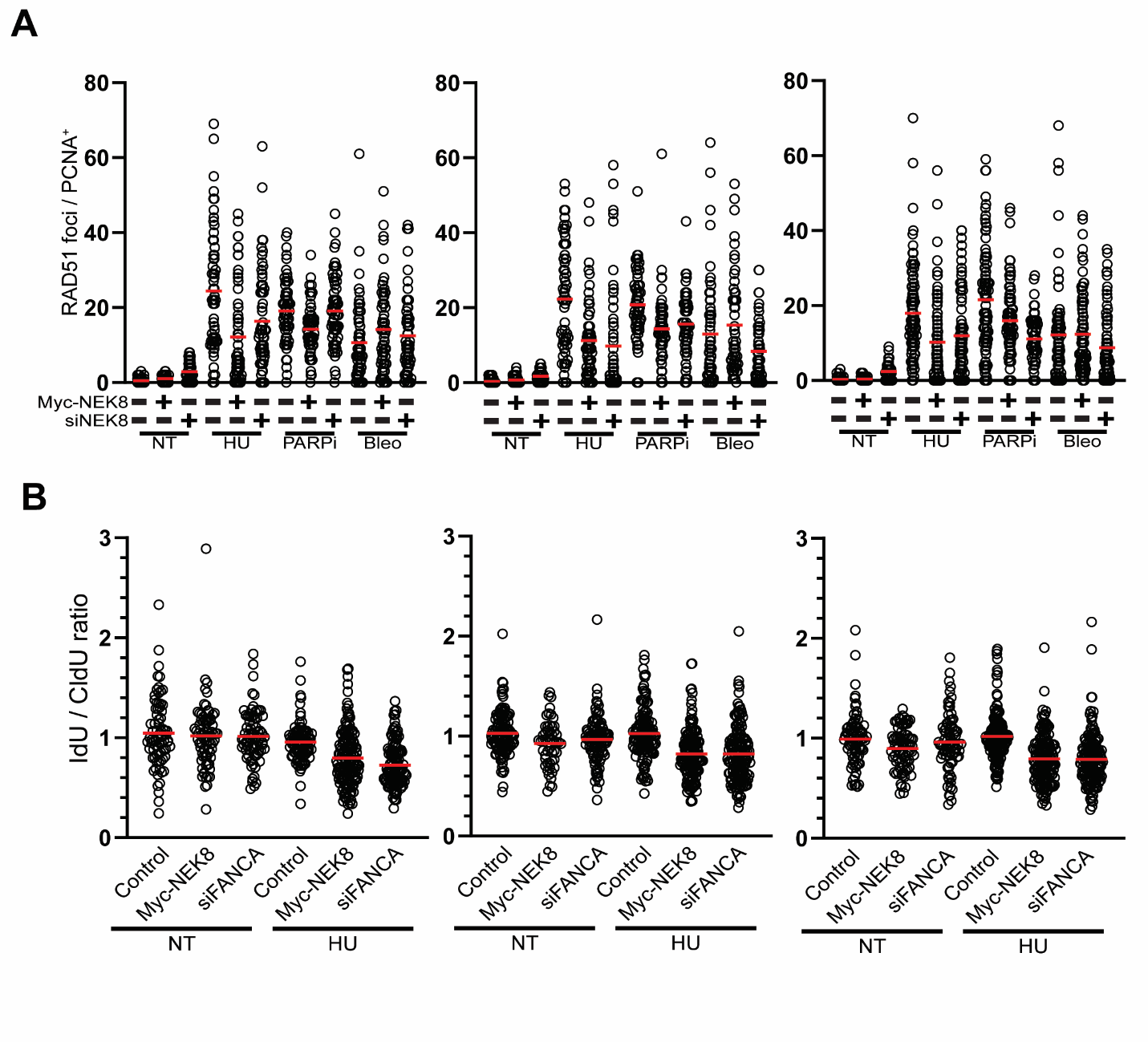


**Figure S2. Individual replicates for dot plots.** (A) Individual replicates for Figure 2B. Individual replicates for Figure 3C.
